# Fire Seasonality, Seasonal Temperature Cues, Dormancy Cycling, and Moisture Availability Mediate Post-fire Germination of Species With Physiological Dormancy

**DOI:** 10.3389/fpls.2021.795711

**Published:** 2021-12-03

**Authors:** Berin D. E. Mackenzie, Tony D. Auld, David A. Keith, Mark K. J. Ooi

**Affiliations:** ^1^Centre for Ecosystem Science, School of Biological, Earth and Environmental Sciences, University of New South Wales, Kensington, NSW, Australia; ^2^Science, Economics and Insights Division, NSW Department of Planning, Industry and Environment, Parramatta, NSW, Australia; ^3^School of Earth, Atmospheric and Life Sciences, University of Wollongong, Wollongong, NSW, Australia; ^4^NSW Bushfire Risk Management Research Hub, Wollongong, NSW, Australia

**Keywords:** fire regime change, fire severity, heat pulse, Rutaceae, seasonal germination niche, smoke, soil seed bank, seedling recruitment

## Abstract

Fire seasonality (the time of year of fire occurrence) has important implications for a wide range of demographic processes in plants, including seedling recruitment. However, the underlying mechanisms of fire-driven recruitment of species with physiological seed dormancy remain poorly understood, limiting effective fire and conservation management, with insights hampered by common methodological practices and complex dormancy and germination requirements. We sought to identify the mechanisms that regulate germination of physiologically dormant species in nature and assess their sensitivity to changes in fire seasonality. We employed a combination of laboratory-based germination trials and burial-retrieval trials in natural populations of seven species of *Boronia* (Rutaceae) to characterize seasonal patterns in dormancy and fire-stimulated germination over a 2-year period and synthesized the observed patterns into a conceptual model of fire seasonality effects on germination. The timing and magnitude of seedling emergence was mediated by seasonal dormancy cycling and seasonal temperature cues, and their interactions with fire seasonality, the degree of soil heating expected during a fire, and the duration of imbibition. Primary dormancy was overcome within 4–10 months’ burial and cycled seasonally. Fire-associated heat and smoke stimulated germination once dormancy was alleviated, with both cues required in combination by some species. For some species, germination was restricted to summer temperatures (a strict seasonal requirement), while others germinated over a broader seasonal range of temperatures but exhibited seasonal preferences through greater responses at warmer or cooler temperatures. The impacts of fires in different seasons on germination can vary in strength and direction, even between sympatric congeners, and are strongly influenced by moisture availability (both the timing of post-fire rainfall and the duration soils stay moist enough for germination). Thus, fire seasonality and fire severity (via its effect on soil heating) are expected to significantly influence post-fire emergence patterns in these species and others with physiological dormancy, often leading to “germination interval squeeze.” Integration of these concepts into current fire management frameworks is urgently required to ensure best-practice conservation. This is especially pertinent given major, ongoing shifts in fire seasonality and rainfall patterns across the globe due to climate change and increasing anthropogenic ignitions.

## Introduction

### Global Changes in Fire Regimes

Fire plays a crucial role in the maintenance of biodiversity in fire-prone ecosystems (He et al., 2019). Terrestrial plants have evolved a variety of life history traits that enable their persistence through recurrent fire by promoting the survival of individuals and/or offsetting mortality via *in situ* recruitment or recolonization (Noble and Slatyer, 1980; Keeley et al., 2011; Keith, 2012). Individual species are adapted to suit certain fire regimes defined by the frequency, intensity, severity, type, and seasonality of fire (Gill, 1975; Pausas and Keeley, 2009). In turn, these components of the fire regime filter the species able to persist at a given site (Pausas et al., 2004; Archibald et al., 2017). Climate change and other anthropogenic activities (e.g., land-use change, ignitions and fire suppression) are driving rapid changes in global fire regimes (Bowman et al., 2009; Krawchuk et al., 2009; Pausas and Keeley, 2021) which is increasing the risk of plant population declines and local extinctions (Kelly et al., 2020). A mechanistic understanding of how individual components of the fire regime affect key demographic processes is crucial for accurately predicting species’ responses (and resilience) to such regime shifts (Menges, 2000; Enright et al., 2014), and is urgently required to underpin effective fire management for biodiversity conservation (Bowman et al., 2020; Nolan et al., 2021).

Fire seasonality (the time of year of fire occurrence) is a relatively understudied element of the fire regime (Whelan, 1995) despite significant recent advances (Miller et al., 2019; Keith et al., 2020). Historically, wildfires have been more prevalent in warmer or drier seasons, when fuel moisture and weather conditions are most conducive to ignition and fire spread (Krawchuk and Moritz, 2011); however, global increases in annual fire weather due to climate change have led to fire seasons in many regions around the world beginning earlier and lasting longer (Westerling et al., 2006; Jolly et al., 2015). In conjunction with greater anthropogenic ignitions, this is increasing the frequency of unseasonal fires (those outside the historical fire season) in the landscape (Le Page et al., 2010; Balch et al., 2017; Bowman et al., 2020). Variation in fire seasonality can adversely affect plant populations through negative impacts on critical life history stages including adult survival and growth, propagule availability, dispersal, and post-fire seedling establishment (Miller et al., 2019; Keith et al., 2020; and references therein). However, supporting evidence for these mechanisms is relatively limited across most climate and vegetation types—in particular, evidence of how altered fire seasonality affects recruitment from soil seed banks of species with innate seasonal germination requirements (Miller et al., 2019; Cao et al., 2020; Tangney et al., 2020).

### Physiological Seed Dormancy

Species with a physiological component to their dormancy [including physiological dormancy (PD) and morpho-physiological dormancy (MPD)] constitute a significant component of the floristic diversity in fire-prone regions (Merritt et al., 2007; Baskin and Baskin, 2014; Collette and Ooi, 2021) and account for a disproportionate number of threatened species in some climate regions (e.g., Collette and Ooi, 2021). However, physiological dormancy remains poorly understood in these ecosystems, with many species reported as difficult to germinate (Merritt et al., 2007). This is largely due to gaps in our knowledge regarding the complex interactions between multiple fire-related cues, and environmental cues, that break dormancy and stimulate germination from soil seed banks (Mackenzie et al., 2016).

**FIGURE 1:**
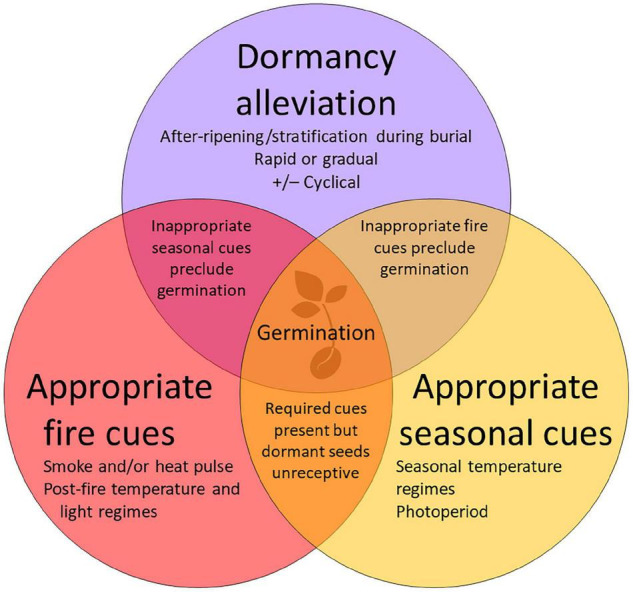
Overlapping conditions required for germination of plant species with physiological seed dormancy in temperate fire-prone ecosystems, given adequate soil moisture. After-ripening and/or stratification are required to overcome primary dormancy in freshly dispersed, mature seeds before they become receptive to one or more fire-related cues required to stimulate germination. Some species also require particular seasonal temperature cues to germinate. The amount and duration of available soil moisture will also influence germination.

Physiological dormancy is overcome in nature by a period of after-ripening and/or stratification during burial (Baskin and Baskin, 2014; Figure 1). In fire-prone ecosystems, fire-associated heat and/or smoke, together with sufficient soil moisture, are required to stimulate germination *once dormancy has been alleviated* and this concentrates seedling emergence in the post-fire environment where there are greater resources and reduced competition (Whelan, 1995). An additional seasonal temperature requirement restricts post-fire germination of some PD species to particular seasons (Ooi et al., 2006; Mackenzie et al., 2016; Collette and Ooi, 2017, 2020) and may lead to delayed post-fire emergence following unseasonal fires (Ooi et al., 2004; Auld and Ooi, 2008; Cao et al., 2020). Loss of PD is progressive, with seeds moving from a dormant state (unresponsive to germination cues) to conditional dormancy (able to germinate over a narrow range of conditions) and finally becoming non-dormant (able to germinate over a wide range of conditions) (Baskin and Baskin, 2014). Some species cycle in and out of dormancy in response to seasonal variation in temperature and soil moisture (Merritt et al., 2007; Baskin and Baskin, 2014), resulting in periodic changes in their receptivity to germination cues (Baker et al., 2005c). Even in strongly seasonal rainfall environments where the availability of soil moisture restricts germination to a particular time of year, there are examples of species with innately seasonal germination (de Lange and Boucher, 1993; Roche et al., 1998; Chia et al., 2016).

### Barriers to Understanding Physiological Dormancy in Fire-Prone Ecosystems

Efforts to understand the seed ecology and germination requirements of PD species in natural populations in fire-prone ecosystems have been hampered by a number of factors, including common practices in laboratory germination trials, and complex and variable interactions between multiple fire-cues and other environmental cues (Table 1). The poor germination of viable seeds frequently reported in *ex situ* trials of PD species is most frequently attributable to application of germination cues to dormant seeds, or the absence of appropriate fire cues and/or seasonal incubation temperatures (Figure 1). Overreliance in seed ecology research on freshly dispersed seeds and/or seeds stored *ex situ* under artificial conditions provides limited reliable insight into the regulation of dormancy and germination in soil seed banks in natural systems. Fresh seeds with PD typically yield poor germination due to high primary dormancy (e.g., Tieu et al., 2001; Ooi et al., 2006; Mackenzie et al., 2016) and are likely to be poor indicators of how seeds in the soil seed bank respond as they after-ripen and undergo stratification and physical deterioration. *Ex situ* storage of seeds may have impacts on dormancy and germination that are difficult to quantify and vary with storage conditions, precluding reliable extrapolation to populations in the field (Baskin et al., 2006).

**TABLE 1:** Common practices impeding our understanding of how dormancy and germination syndromes regulate post-fire emergence of species with physiological seed dormancy (PD) in fire-prone ecosystems and some practical solutions.

Practice	Potential consequences	Solution
Use of seeds stored *ex situ* under artificial conditions.	Unknown temporal and storage effects on dormancy preclude reliable extrapolation to natural populations.^a^ Not an issue if primary aim is maximizing germination to generate seedlings for *ex situ* conservation or horticultural purposes.^a^	Use freshly dispersed seeds as soon as possible after collection for germination ecology studies aimed at understanding regulation of dormancy and germination in natural systems.^a^
Use of freshly dispersed seeds to predict germination responses of soil seed banks.	Germination often poor due to high primary dormancy. Responses unlikely to represent those of seeds in the soil seed bank where dormancy has been partially or fully alleviated.^b^	Follow up with an *in situ* seed burial-retrieval trial to characterize temporal (seasonal) patterns in dormancy alleviation and germination.
Application of germination cues to dormant seeds.	Poor germination and potentially erroneous conclusions about cue inefficacy if cues that break PD (including heat and smoke) are not distinguished from those that stimulate germination once dormancy is broken.^b,c,d,e,f^	Maintain the distinction between mechanisms of dormancy alleviation (after-ripening, stratification) and cues that stimulate germination once dormancy is overcome when interpreting the causes of poor germination.^b,c,d,e,f^
Overlooking fire-associated heat as a potential germination cue.	Poor germination and failure to identify species with a heat response or heat requirement (this includes members of a diverse range of plant families).	Include a heat pulse in studies of fire-stimulated germination of species with unknown germination syndromes.
Application of fire cues such as heat and smoke in isolation from one another.	Poor germination and failure to identify important interactions and species with obligate germination requirements for two or more fire cues in combination.	Investigate factorial combinations of fire cues.
Overlooking certain seasonal temperatures or application of inappropriate diurnal incubation regimes.	Poor germination and failure to identify species with seasonal germination requirements.	Include a full complement of seasonal incubation temperatures appropriate to the study region, noting that fresh seeds are likely to germinate over a narrower range of temperatures than seeds in the soil seed bank. Avoid constant temperatures and continuous light.^a^
Extended duration of germination trials.	Potential alleviation of dormancy via stratification as the trial progresses, overinflating germination response measurements.	Limit trial length to the plausible duration of continuous seed imbibition in natural populations. This is usually poorly known and will vary seasonally so reporting temporal patterns in germination in addition to final cumulative germination is essential.
Overlooking the role of environmental cues such as light and soil moisture.	Poor germination of, and failure to identify, species sensitive to these cues, or, alternatively, overinflated germination in extended trials with continuous moisture availability.	Examine interactive effects of light and/or soil moisture availability.^g^

*^a^Baskin et al. (2006).*

*^b^Baskin and Baskin (2014).*

*^c^Vleeshouwers et al. (1995).*

*^d^Merritt et al. (2007).*

*^e^Thompson and Ooi (2010).*

*^f^Thompson and Ooi (2012).*

*^g^Thomas et al. (2010).*

A lack of data on the combined effects of multiple fire-related cues and environmental cues on germination of PD species is a key knowledge gap. The positive effect of smoke on germination of PD species is well-documented (Dixon et al., 1995; Keeley and Fotheringham, 1998; Brown and Botha, 2004; Moreira et al., 2010); however, as a result, the importance of the heat pulse associated with the passage of fire is an often-overlooked cue in species with this type of dormancy. This is despite a growing number of reports of positive responses to a heat pulse in a diverse range of families with PD [e.g., Apiaceae (Baker et al., 2005c), Ericaceae (Moreira et al., 2010), Lamiaceae (Kazancı and Tavşanoǧlu, 2019); Myrtaceae (Auld and Ooi, 2009), and Rutaceae (Mackenzie et al., 2016)]. Where both cues have been investigated in combination, responses are highly variable and species-specific, ranging from neutral to additive (Keith, 1997; Kenny, 2000; Newton et al., 2021), unitive (both cues required (Thomas et al., 2007; Mackenzie et al., 2016; Collette and Ooi, 2020); synergistic (Gilmour et al., 2000; Baker et al., 2005a); and negative (a heat pulse inhibits the smoke response without loss of viability; Keeley and Fotheringham, 1998; Collette and Ooi, 2017). Potential interactions between fire cues and environmental cues such as light (Bell, 1994; Gilmour et al., 2000; Collette and Ooi, 2017) and moisture (Thomas et al., 2010) create additional complexity.

The important role that seasonal temperature cues play in regulating fire-stimulated germination of many PD species has been recognized only relatively recently (Ooi et al., 2006; Mackenzie et al., 2016; Collette and Ooi, 2017, 2020; Hodges et al., 2019). Very few studies have investigated the effect of fire-related germination cues such as heat and smoke in combination with a full complement of seasonal temperatures, limiting our understanding of the germination ecology of PD species in temperate fire-prone regions and predictions of the impacts of altered fire regimes on recruitment. In particular, major shifts in the seasonal occurrence of fires pose a threat to PD species with seasonal germination requirements where seedling emergence is restricted to a particular time of year regardless of the timing of fire occurrence (Ooi et al., 2004). Thus, seedling emergence can be delayed by up to 12 months following fires in certain seasons and may also be diminished in magnitude where such delays reduce or negate the efficacy of fire-related cues. This can have adverse effects on establishment success and subsequent plant performance (Ooi, 2019). However, field evidence for this phenomenon is currently very limited (Miller et al., 2019).

Here, we examine mechanisms with the potential to drive seasonal post-fire emergence patterns in PD species within members of the Rutaceae, an important cosmopolitan plant family and one of the most significant families in fire-prone temperate Australia (Collette and Ooi, 2021). Like many other PD species from fire-prone regions, Rutaceae are often reported as difficult to germinate (Brown and Botha, 2004; Martyn et al., 2009; Collette and Ooi, 2020); however, few studies have applied cue combinations that seeds experience in natural populations to viable, non-dormant seeds (Auld, 2001) and, until recently, none had done so in conjunction with a full a complement of seasonal temperatures (Mackenzie et al., 2016; Collette and Ooi, 2017). A poor understanding of the seed and fire ecology of Australian Rutaceae also limits effective management and conservation of this family which contains large numbers of rare and nationally threatened species (Auld, 2001).

We investigated the mechanisms of fire-driven recruitment from soil seed banks in species with PD using *Boronia*, the largest Australian Rutaceae genus, as a case study with the dual aims of addressing knowledge gaps in the ecology of this important plant family and of improving understanding of PD in fire-prone ecosystems generally. We employed a combination of laboratory-based germination trials and burial-retrieval trials in natural populations to characterize seasonal patterns in dormancy and fire-stimulated germination over a 2-year period. Our primary aims were to:

i) identify species with seasonal temperature requirements for germination,
ii) quantify seasonal changes in dormancy, and
iii) develop mechanistic models of the effect of fire seasonality on seedling emergence phenology and magnitude of species with PD.

## Materials and Methods

### Study Area and Study Species

**FIGURE 2:**
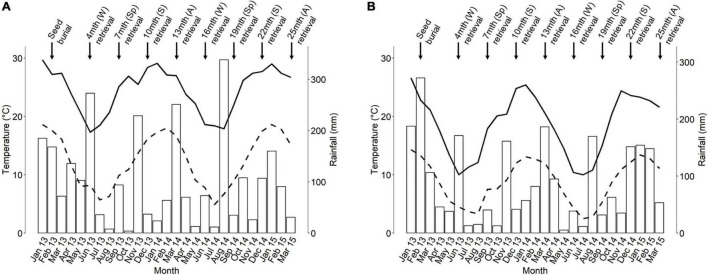
Indicative monthly rainfall (white bars) and mean monthly maximum (solid line) and minimum (dashed line) ambient temperature at the **(A)** coastal and **(B)** upper tablelands study sites during the study period. Data are courtesy of the Australian Government Bureau of Meteorology (2021). Temperature and rainfall data in **(A)** are from the Holsworthy Aerodrome and Audley Royal National Park weather stations, respectively. All data in **(B)** are from the Mt Boyce weather station. S, summer; A, autumn; W, winter; Sp, spring.

The study was undertaken in the Sydney region of south-eastern Australia, with the approval of the New South Wales Office of Environment and Heritage (Scientific License No. SL101105). The regional climate is temperate with no dry season, according to the Köppen classification (Stern et al., 2000). Rainfall is aseasonal (Figure 2), meaning that germination can occur year-round cf. Mediterranean-type climates where dry summers usually restrict germination to the cooler, wetter months.

Seven species of *Boronia* were selected for study on the basis of their fecundity, overlap in flowering phenology, and populations within the study region that were large enough to provide the requisite quantities of seed. The study species are all shrubs from fire-prone heaths and woodlands and comprise a mixture of obligate seeders and resprouters, rare and common species, and different evolutionary lineages (sections) within the genus (Table 2). Further details on their seed morphology and the locations of the study sites are provided in Mackenzie et al. (2016).

**TABLE 2:** Study species.

Species	Section^a^	Habitat^b^	Fire response	Regional significance^b^
*Boronia anemonifolia* subsp. *anemonifolia* A.Cunn	*Cyanothamnus*	Among rocks in open forest and heath	Resprouts^b,c^ but can be variable^d^	Widespread on coast and ranges
*Boronia floribunda* Sieber ex Rchb.	*Boronia*	Ridgetops and rock outcrops in open forest and heath	Resprouts^b,c,d^ but can be variable^d^	Local endemic
*Boronia fraseri* Hook.	*Valvatae*	Gullies in moist eucalypt open forest	Killed by fire^c^	Rare local endemic
*Boronia ledifolia* (Vent.) DC.	*Valvatae*	Ridges and rocky outcrops in woodland	Killed by fire^b,c,d^	Widespread on coast and ranges
*Boronia pinnata* Sm.	*Boronia*	Ridges and plateaus in eucalypt forest and heath	Resprouts^b,c,d^ but can be variable^c,d^	Chiefly coastal
*Boronia serrulata* Sm.	*Boronia*	Rock outcrops and platforms in moist heath and woodland	Killed by fire^b,c,d^	Rare local endemic
*Boronia thujona* A.R. Penfold and M.B.Welch	*Boronia*	Gullies, creeks, cliff lines in moist eucalypt open forest	Killed by fire^b,c^	Northern limit of distribution

*^a^Duretto et al. (2013).*

*^b^Benson and McDougall (2001).*

*^c^NSW Office of Environment and Heritage (2014).*

*^d^B. D. E. Mackenzie (pers. obs).*

### Seed Collection and Field Burial Trials

Fruits are ballistic at maturity, so seeds were captured at dispersal using light-weight polypropylene bags tied around fruiting branches. Seeds were stored in the laboratory at ambient temperature (c. 20–25°C) for 4 weeks [*B. anemonifolia* subsp. *anemonifolia* (hereafter, *B. anemonifolia*), *B. fraseri*, *B. pinnata*, *B. serrulata*] to 7 weeks (*B. floribunda*, *B. ledifolia*, *B. thujona*) prior to the commencement of burial trials.

Replicate burial plots (three for *B. thujona* and four for all other species) were established in mid-summer (late January to early February) at the original seed collection site for each species and positioned up to 15 m apart amongst random stands of mature *Boronia* individuals. Replicate batches of seeds (25 seeds each for *B. ledifolia*; 24 seeds each for *B. thujona;* 20 seeds each for *B. anemonifolia*, *B. floribunda*, *B. fraseri* and *B. serrulata*; and 18 seeds each for *B. pinnata*) were mixed with a spoonful of local topsoil that had been finely sifted to remove any pre-existing seeds. The seed/soil mixture was placed inside 10 cm lengths of nylon stocking and sealed with a knot at each end. This enabled free movement of soil moisture and limited the volume of soil to be searched during later seed recovery. Additional protection from disturbance by animals was provided by placing bags inside durable fiberglass pockets made of 2 mm insect mesh and measuring 5 cm × 10 cm. Replicate pockets were arranged in a contiguous grid and buried 1–2 cm below the soil surface followed by reinstatement of the surface litter.

Commencing in winter (June), random samples of bags were exhumed from replicate burial plots at the beginning of each season for up to 2 years (i.e., at 4, 7, 10, 13, 16, 19, 22, and 25 months post-burial). Retrievals of *B. anemonifolia* were limited to 4, 7, 10, and 13 months due to lower seed availability. The contents of retrieved bags were air-dried in the laboratory for 1–2 weeks and then searched for seeds or seed remains. Recovered seeds were classified as “empty/unfilled,” “dead/inviable,” “germinated,” or “ungerminated and intact,” with the latter seeds subjected to germination trials.

### Seasonal Germination Trials

Seasonal germination trials were conducted over a 2-year period with the primary aims of (i) measuring seasonal changes in the state of dormancy of buried seeds and (ii) predicting germination responses to fires in different seasons. Seeds buried for 4–13 months were subjected to factorial combinations of fire-associated heat (10 min exposure to 80°C), smoke (10 min exposure to aerosol smoke generated from burning vegetation), and seasonal incubation temperatures (approximating summer, autumn/spring and winter at the study sites), following the methodology of Mackenzie et al. (2016) and using the same species and seed lots. Two species with more limited seed availability (*B*. *pinnata* and *B. thujona*) were subjected to factorial combinations of smoke and seasonal temperatures only (Supplementary Material 1). Germination trials ran for 14 weeks, approximating the length of a season.

Seeds buried for 16–25 months were subjected to species-specific cue combinations that were found to maximize germination during their first year of burial (Table 3). Treatments representing fires in different seasons were also applied and these comprised the most effective combination of fire cues (a heat pulse and/or smoke) for each species followed by incubation at whichever seasonal temperature corresponded with field conditions at the time of retrieval.

**TABLE 3:** The most effective combinations of germination cues for seven species of *Boronia* (Rutaceae) from south-eastern Australia observed during the first year of a burial trial using freshly collected seeds.

Section/Species	Cue combinations
***Boronia***	
*B. floribunda*	S + winter, HS + winter
*B. pinnata*	S + summer
*B. serrulata*	S + winter, HS + autumn/spring
*B. thujona*	S + summer, S + autumn/spring
***Cyanothamnus***	
*B. anemonifolia*	HS + summer
***Valvatae***	
*B. fraseri*	H + summer, HS + summer
*B. ledifolia*	H + summer, HS + summer

*Fire cue treatments include a heat pulse (H), smoke (S), and a heat pulse plus smoke (HS). Seasons refer to seasonal incubation temperatures. Refer to main text for details.*

### Analytical Methods

#### Measuring Dormancy

**FIGURE 3:**
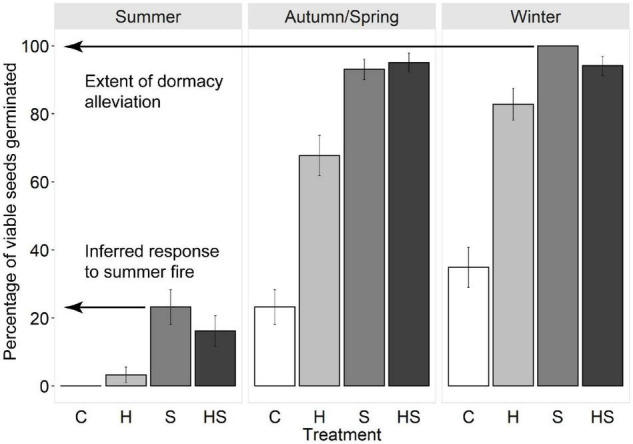
Illustration of how seasonal changes in the state of dormancy and germination responses to fires in different seasons were inferred in the present study. In this example, seeds of *Boronia floribunda* were exhumed in early summer after 10 months’ burial and were subjected to factorial combinations of fire cues (C, control; H, heat pulse; S, smoke; HS, combined heat pulse plus smoke treatment) and incubated at one of three seasonal temperatures (top bar: summer, autumn/spring, winter). The minimum percentage of seeds in a non-dormant state after 10 months’ burial was inferred from the maximum germination response observed across all treatments (here, 100% after 6 weeks’ incubation). The inferred germination response to a summer fire *in situ* was taken as the maximum germination observed in response to any combination of fire cues at summer incubation temperatures (here, c. 24% after 6 weeks’ incubation).

As dormancy cannot be directly measured, the degree of germinability was used to infer the state of dormancy of buried seeds (Vleeshouwers et al., 1995). Seeds that germinate are, by definition, in a non-dormant state and fire-associated heat and smoke do not break PD—they only stimulate germination once dormancy has been alleviated (Baker et al., 2005b,c; Merritt et al., 2007; Thompson and Ooi, 2010; Mackenzie et al., 2016). Hence, at each seasonal retrieval, a *minimum* estimate of the proportion of seeds in a non-dormant state was inferred from the *maximum* germination response observed across all treatments (Figure 3). This approach was robust to an incubator failure at 13 months which limited data for *B. fraseri* and *B. ledifolia* (see Supplementary Material 2).

#### Measuring Seasonal Responses to Fire Cues

Following each seasonal retrieval of buried seeds, the maximum germination observed in response to any combination of fire cues at the incubation temperature corresponding with field conditions at the time of retrieval was used to infer the likely response of seeds *in situ* to a fire occurring in that season (Figures 2, 3). Clear differences in responses between seasons, particularly significant germination vs. no germination, did not require inferential statistics.

## Results

### Seasonal Patterns in Dormancy

**FIGURE 4:**
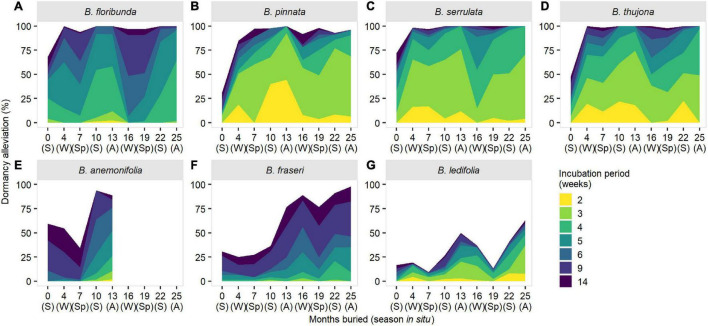
Seasonal changes in dormancy (as measured by maximum germination response) and the effect of incubation period for seven species of *Boronia* (Rutaceae) from south-eastern Australia. S, summer; A, autumn; W, winter; Sp, spring. Species are arranged by sections within the genus: **(A–D)** *Boronia*; **(E)** *Cyanothamnus*; **(F,G)** *Valvatae*. *Boronia anemonifolia* was studied for 1 year only due to limited seed availability. Data for *B. fraseri* and *B. ledifolia* at 13 months have been imputed (refer to Supplementary Material 2 for details).

Seasonal patterns in dormancy were evident in all seven species and broad patterns were reasonably consistent within sections in the genus (Figure 4). Dormancy estimation was markedly affected by incubation period—a proxy for the duration of sufficient soil moisture for imbibition and germination *in situ*—with longer incubation promoting greater loss of dormancy due to stratification effects (Figure 4). Seasonal patterns in dormancy were persistent (i.e., independent of incubation period) in sections *Cyanothamnus* and *Valvatae* (Figures 4E–G) but became less pronounced or disappeared altogether in section *Boronia* after 6–14 weeks’ incubation (Figures 4A–D).

Primary dormancy was highest in sections *Valvatae* and *Cyanothamnus*, and variable in section *Boronia*. Dormancy loss was fastest and greatest in section *Boronia* (substantial alleviation within 4 months of burial/the first winter in the seed bank) while dormancy remained high in other species until the second summer (10 months’ burial) or autumn (13 months’ burial) (Figure 4). Dormancy was almost entirely (94–100%) overcome for all species at some point during the 2-year study; however, *B. ledifolia* proved an exception with a maximum detectable dormancy loss of 64% (Figure 4).

Section *Boronia* species were characterized by pronounced reductions in dormancy alleviation in winter or winter-spring (at least in the second in the year of burial) with peak dormancy loss (maximum receptivity to germination cues) in spring-summer (Figures 4A–D). These reductions coincided with spring in section *Valvatae* species with peak dormancy alleviation in winter and summer (*B. fraseri*) and autumn (*B. ledifolia*) (Figures 4F,G). A similar pattern of peak receptivity was apparent in *B. anemonifolia* (Figure 4E); however, more than 1 year of data is required to confirm if dormancy is cyclical in this species.

### Seasonal Patterns in Responses to Fire Cues

**FIGURE 5:**
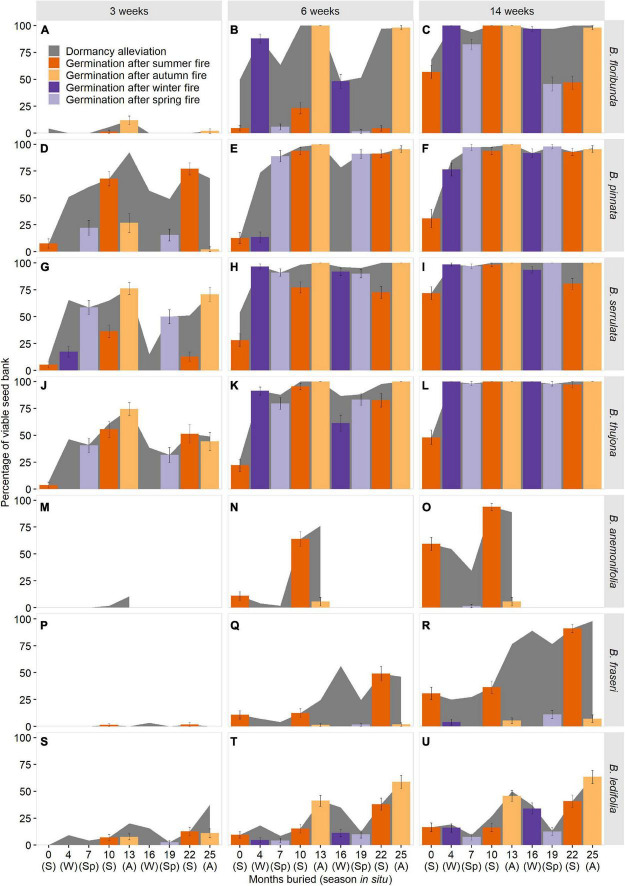
Predicted germination in response to fires in different seasons for seven species of *Boronia* (Rutaceae) from south-eastern Australia as function of incubation period. S, summer; A, autumn; W, winter; Sp, spring. Background shading indicates the proportion of the viable seed bank in a non-dormant or conditionally dormant state and able to respond to combinations of seasonal temperatures and fire-related germination cues. Species are arranged by sections within the genus: *Boronia* **(A–L)**; *Cyanothamnus* **(M–O)**; and *Valvatae* **(P–U)**. *Boronia anemonifolia* was studied for 1 year only due to limited seed availability. Dormancy estimates for *B. fraseri* and *B. ledifolia* at 13 months have been imputed (refer to Supplementary Material 2 for details).

Seasonal fire treatments had a profound effect on the timing and magnitude of germination in all seven species (Figure 5). As per dormancy estimation, seasonal patterns in fire-stimulated germination were highly influenced by incubation period—and, by inference, the duration of soil moisture availability *in situ*—with longer wet intervals leading to marked increases in germination for all species, but not across all seasons for every species. Three section *Boronia* species (*B. pinnata*, *B. serrulata*, *B. thujona*) were able to germinate to appreciable levels (≥50%) in response to treatments representative of a fire within 3 weeks of incubation in at least one season (Figures 5D,G,J), while other species required longer periods of imbibition to respond.

High levels of dormancy limited germination of some species in certain seasons (e.g., Figures 5J,N,Q,U). However, dormancy loss did not always equate to germination (e.g., Figures 5B,D,O,R), with seasonal temperature requirements, or temperature-dependent germination speed (seasonal temperature preferences *sensu* Mackenzie et al., 2016), constraining the timing and magnitude of the response.

An obligate germination requirement for summer temperatures precluded germination of *B. anemonifolia* and *B. fraseri* in response to treatments representative of fire in autumn, winter, and spring, regardless of the extent of dormancy alleviation (Figures 5M–R). Other species were able to germinate in response to fire treatments across a broader range of seasons, with the seasonal range and magnitude of responses increasing with the duration of incubation.

Over shorter periods, non-trivial germination of *B. ledifolia* was restricted to treatments representing autumn fires and—in the second year of burial—a summer fire (Figures 5S,T). Extended incubation increased the response to treatments representing winter fires treatments (a function of temperature-dependent germination rates; germination of *B. ledifolia* is slowest at winter temperatures but can occur given adequate time); however, treatments representing spring fires continued to result in negligible germination due to high seasonal dormancy (Figure 5U). In section *Boronia*, longer incubation increased germination responses to a broader range of seasonal fire treatments through a combination of increased dormancy alleviation (slower in *B. floribunda* than the other three species) and greater time available for slower germination at certain seasonal temperatures to occur (i.e., cooler seasons for *B. pinnata* and warmer seasons for the remaining species; Figures 5A–L).

### Conceptual Model of Seasonal Germination

**FIGURE 6:**
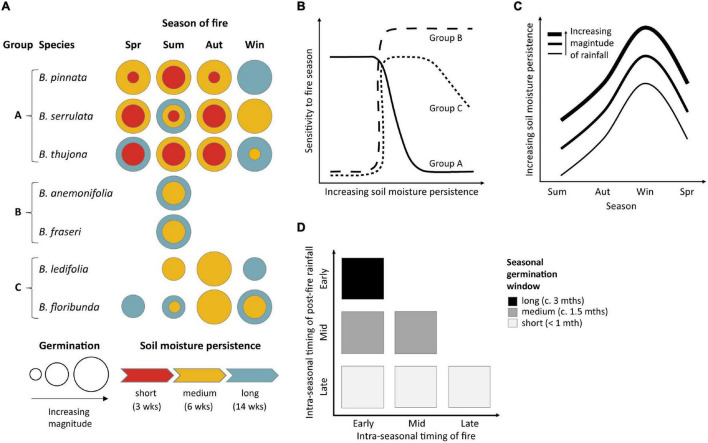
Conceptual models of how the seasonal timing of fire and soil moisture availability affect germination of seven species of *Boronia* (Rutaceae) from south-eastern Australia. **(A)** Functional response groups defined by predicted effects of fire seasonality and soil moisture persistence on germination timing and magnitude. Spr, spring; Sum, summer; Aut, autumn; Win, winter; blank spaces – no germination response with 14 weeks. Where longer durations did not increase germination only the color for the shorter duration is presented. **(B)** Sensitivity to fire seasonality as a function of soil moisture persistence for the response groups identified in **(A)**. **(C)** Seasonal variation in the availability of soil moisture for germination and the effect of rainfall magnitude. Soil moisture persistence increases with greater precipitation (different curves) and is inversely related to evapotranspiration which increases in warmer months (Supplementary Material 3). **(D)** Influence of within-season timing of fire and subsequent rainfall on the relative length of the germination window in the season of fire occurrence.

A conceptual model (Figure 6) illustrates the importance of both the seasonal timing of fire and soil moisture availability in expected post-fire germination patterns. The study species can be divided into three broad functional response groups based on their germination speed and predicted emergence patterns (Figure 6A). Species with slower germination and more restricted seasonal temperature requirements are likely to be more sensitive to fires in different seasons (Figure 6B, Groups B and C), although seasonal differences in germination are expected to decrease with increasing availability of soil moisture for species capable of germination over broader seasonal ranges (Figures 6A,B, Groups A and C). Interactions between seasonal differences in soil moisture persistence and temperature-dependent germination speed are expected to reduce germination of some species following fires in warmer seasons due to increasingly transient soil moisture (Figure 6C; see also Supplementary Material 3). We term this phenomenon “germination interval squeeze.” Mid- to late-season fires and/or lags in post-fire rainfall further contribute to germination interval squeeze by significantly shortening the potential window of post-fire imbibition and germination within the season of fire occurrence (Figure 6D). Slower-to-germinate species and/or those with narrower seasonal requirements are most sensitive to this type of interval squeeze—especially those with obligate germination requirements for summer temperatures (Figure 6A, Group B) where late summer fires and/or delayed rainfall may preclude germination for up to 9–10 months until the following summer.

## Discussion

### Key Drivers of Seedling Emergence Phenology and Magnitude

Seasonal temperatures play an important role in the germination of all seven species investigated, restricting germination of some species to a single season, and concentrating germination of others in warmer or cooler parts of the year. Our models indicate that the timing and magnitude of seedling emergence in natural systems are mediated by seasonal dormancy cycling and seasonal temperature cues, and their interactions with the seasonal timing of fire and soil moisture availability. Fire seasonality effects on emergence patterns vary in their strength and direction, even between sympatric congeners, and are strongly influenced by soil moisture availability (both the timing of availability in relation to fire and the duration that moist soils persist) which is expected to vary seasonally. This greatly increases the stochasticity of seedling recruitment of PD species in these ecosystems due to natural variability in post-fire rainfall and fire ignitions, with climate change and increasing anthropogenic ignitions introducing further complexity and variation via impacts on rainfall patterns and shifting fire seasonality.

### Fire Seasonality Effects

Delayed or reduced germination is expected to occur following fires in one or more seasons for all seven species due to mismatches between their dormancy and germination phenology and the seasonal timing of fire. Effects are likely to be exacerbated where soil moisture availability is short-lived and/or delayed post-fire (“germination interval squeeze”; Figure 6). Slow-to-germinate species with narrow seasonal tolerances are the most vulnerable to potential impacts of altered fire seasonality on emergence patterns, especially where germination is cued to warmer months where soil moisture is more limiting. Obligate seeders such as the rare *B. fraseri* are most at risk due to reliance on post-fire seedling recruitment for population recovery and persistence. However, resprouters with variable capacity for post-fire vegetative recovery such as *B. anemonifolia* are also susceptible.

Altered fire seasonality, which we define for the study region as fires outside late spring to summer, is predicted to have varied effects on PD species. For the majority of species, unseasonal fires in one or more seasons are expected to delay and/or diminish germination relative to summer fires (Figures 5, 6A). However, our models suggest that certain out-of-season fires may actually reduce the time to emergence for some species. For example, fires outside of summer may increase and accelerate germination of *B. serrulata*; autumn fires may increase and accelerate germination of *B. ledifolia*; and germination of *B. floribunda* may be accelerated by autumn and winter fires. Given that Miller et al. (2019) found no positive demographic effects of altered fire seasonality in their global review, it will be of interest and significance to note whether or not faster emergence of these species following certain unseasonal fires (if it occurs under field conditions as predicted) does in fact lead to increased recruitment success.

Earlier emergence is regarded as advantageous in non-fire prone ecosystems (Verdú and Traveset, 2005). However, there is a relative paucity of quantitative data from fire-prone ecosystems in regions with aseasonal rainfall concerning the consequences of variation in post-fire emergence timing (on a scale of days and weeks to months and/or years) for successful seedling establishment and subsequent plant performance—especially for geosporous species. Nonetheless, late germinants are expected to be disadvantaged if they miss the peak post-fire resource flush associated with ash deposition, or if they are exposed to competition from more rapidly established vegetative and seedling regenerators.

Finally, the effect of increasing time since fire on the stimulatory efficacy of fire-associated heat and smoke on germination of PD species has received little research attention. Where post-fire germination is delayed due to inappropriate seasonal temperatures and/or delayed post-fire rainfall, germination magnitude might be unaffected, diminished, or completely nullified depending on the extent of the delay. The mechanism by which fire-associated heat promotes germination of PD species remains unknown (Mackenzie et al., 2016); however, the active constituents in smoke are water-soluble (Flematti et al., 2004) and, over time, will presumably be leached out of the topsoil where most of the seed bank resides (although Preston and Baldwin (1999) suggest the smoke cue can persist in soil for 7 years or longer). Delayed post-fire rainfall may delay exposure to the smoke cue relative to the heat pulse cue (the latter being coupled with fire passage) which could be important for species that require both cues to germinate. Conversely, smoke may leach into the soil in gaseous form immediately following fire and later in aqueous form via rainfall, and where rainfall is aseasonal as in our study region, delays between fire passage and post-fire rainfall will most often be minimal, even following unseasonal fires. Nevertheless, the effect of time since fire on fire-cue efficacy warrants further investigation in the study of fire seasonality effects on seedling emergence patterns in PD species.

### Implications for Fire Management and Plant Conservation

As well as mismatches between seasonal occurrence of fire and germination phenology, fire seasonality may also influence the magnitude of post-fire seedling emergence via seasonal trends in soil moisture and fire severity. Higher moisture content in seeds lowers their lethal temperature thresholds (Tangney et al., 2018); hence, fires occurring in cooler seasons where soil moisture tends to be higher might be expected to increase seed mortality and reduce seedling emergence (Le Fer and Parker, 2005), depending on fire severity. Furthermore, cool-season fires tend to have lower fire severity and hence, lower depth and duration of soil heating due to reduced consumption of fine fuels (Bradstock and Auld, 1995). Dissipation of thermal energy by soil moisture (Stoof et al., 2011) is also generally greater in cooler seasons. This is especially relevant to species where a heat pulse is an obligate germination requirement (e.g., *B. fraseri* and *B. ledifolia* require a heat pulse in combination with smoke) or is required to maximize germination (e.g., *B. anemonifolia*). Contrary to its well-established role in the germination of hard-seeded (physically dormant) species (Jeffrey et al., 1988; Auld and O’Connell, 1991; Keeley, 1991; Reyes and Trabaud, 2009), fire-associated heat has been largely overlooked as a germination cue in PD species due to a focus on the widespread stimulatory effects of smoke (Dixon et al., 1995; Brown and Botha, 2004; Tormo et al., 2014). However, positive responses to heat pulses, including interactions between heat and smoke, have been reported in PD species across a wide range of plant families (see Introduction; reviewed by Mackenzie et al., 2016). Hence, the degree of soil heating during fires may influence recruitment of many PD species and fire severity is thus an important consideration in fire management for their conservation.

### Improving Understanding of *in situ* Germination

Reporting temporal patterns in germination is a key way in which to improve the ecological utility of laboratory-based germination studies. Germination responses are routinely censused at multiple timepoints over the course of a trial yet the majority of studies only present final total cumulative germination after a given number of weeks or months of continuous imbibition and incubation. This is sufficient where the primary aim is on maximizing germination (e.g., for horticultural or *ex situ* conservation/restoration purposes). However, for ecological studies that seek to understand and predict species responses *in situ*, data on temporal patterns—including the time to onset of germination and subsequent germination speed and synchrony—are crucial given that moisture and temperature conditions amenable to germination are temporally limited. As demonstrated here, the duration of imbibition has a profound effect on dormancy and germination responses, and studies with an ecological focus should ensure that the length of germination trials and the incubation periods for which results are reported are ecologically plausible, i.e., reflect the intervals and temperatures over which seeds in the upper layers of the soil are likely to remain continuously imbibed in the post-fire environment. Three months seems an appropriate maximum for most temperate fire-prone ecosystems; however, continuous periods of seed imbibition are likely to be much shorter than this in the absence of high or extended rainfall events, especially in warmer seasons. Further studies quantifying *in situ* variation in soil moisture availability in different seasons (e.g., Merritt et al., 2007) across a range of habitat types, and the water potentials across which germination can occur (e.g., Thomas et al., 2010), would greatly improve extrapolation of laboratory-based germination studies to natural populations.

Greater use of seeds aged naturally *in situ* in experimental studies would also significantly improve ecological understanding. Artificial storage may confound natural patterns in seed dormancy and germination responses in unpredictable ways (Baskin et al., 2006), precluding reliable inferences about the responses of soil seed banks *in situ*. Where primary dormancy is low, the responses of fresh seeds to combinations of fire and seasonal temperature cues may help to identify PD species with seasonally sensitive germination. However, as demonstrated here, fresh seed responses are of limited use in predicting the responses of buried seeds due to the effects of burial duration (including seed age) and seasonal cycles in dormancy and germination responses.

Burial trials over long durations are required to accurately characterize soil seed bank dynamics. We followed single cohorts of seeds for 2 years and observed variation in dormancy and germination responses to certain cue combinations between years in some species (Figures 4, 5). A longer experiment could have provided further insights. The distribution of seed ages in the seed bank is unknown for these species but older seeds might be expected to exhibit more stable annual patterns, among other differences. Studies of shorter duration may also be more prone to stochastic events including temperature or rainfall aberrations due to heatwaves, droughts, and/or extreme rainfall events.

## Conclusion

This study has highlighted seasonal temperature requirements and seasonal patterns in dormancy cycling and moisture availability as key drivers of fire-stimulated germination of PD species in fire-prone ecosystems. The mechanistic models proposed here predict significant effects of fire seasonality, fire severity, and soil moisture duration on post-fire emergence patterns, including the increasing risk of “germination interval squeeze,” and call for a more sophisticated and wholistic approach to fire management that explicitly addresses these fire regime and environmental components in addition to the effects of fire frequency (Bradstock and Kenny, 2003). This is urgently required given strong evidence from across the globe of shifting and broadening fire seasonality and reduced seed banks due to interval squeeze (Enright et al., 2015).

The models presented here require field validation and the demographic consequences of any realized delays or reductions in seedling emergence following out-of-season fires need to be evaluated in terms of growth, fecundity, and longevity of recruits. Evidence of adverse effects of delayed emergence on post-fire seedling establishment following unseasonal fires is beginning to emerge (Risberg and Granström, 2009; Ooi, 2010) but is currently scarce. Field studies of post-fire recruitment involving replicated fires across a range of seasons and sites are vital to establish the magnitude of the threat and the extent to which different species (especially rare or threatened taxa), plant families and functional groups are resilient. In the meantime, a precautionary approach to fire management that limits the occurrence of (successive) out-of-season fires is likely to benefit the greatest diversity of species.

## Data Availability Statement

The original contributions presented in the study are included in the article/Supplementary Material, further inquiries can be directed to the corresponding author/s.

## Author Contributions

BM conducted the field and experimental work, analyzed the data, and led the writing of the manuscript. All authors conceived the ideas, designed the research, contributed critically to the drafts, and gave final approval for publication.

## Conflict of Interest

The authors declare that the research was conducted in the absence of any commercial or financial relationships that could be construed as a potential conflict of interest.

## Publisher’s Note

All claims expressed in this article are solely those of the authors and do not necessarily represent those of their affiliated organizations, or those of the publisher, the editors and the reviewers. Any product that may be evaluated in this article, or claim that may be made by its manufacturer, is not guaranteed or endorsed by the publisher.
