# Supplementary material for: Fire Seasonality, Seasonal Temperature Cues, Dormancy Cycling, and Moisture Availability Mediate Post-fire Germination of Species With Physiological Dormancy
Source: Front Plant Sci. 2021 Dec 3;12:795711. doi: 10.3389/fpls.2021.795711 (PMC8678276; doi:10.3389/fpls.2021.795711)
Supplement: Supplementary file 1 [file Data_Sheet_1.zip › Supplementary Material 1 - Germination treatments.docx]

Supplementary Material

**Fire seasonality, seasonal temperature cues, dormancy cycling, and moisture availability mediate post-fire germination of species with physiological dormancy**

Berin D. E. Mackenzie*, Tony D. Auld, David A. Keith and Mark K. J. Ooi

* Corresponding Author: [berin.mackenzie@environment.nsw.gov.au](mailto:berin.mackenzie@environment.nsw.gov.au) (B. D. E. Mackenzie)

**Supplementary Material 1. Germination treatments applied during the study.**

**Supplementary Table 1.** Combinations of fire-related cues and seasonal incubation temperatures applied during a two-year germination study involving buried seeds of seven species of *Boronia* from southeastern Australia. Treatments are: control (C); a heat pulse (H); smoke (S); and a heat pulse plus smoke (HS). Refer to main text for details.

| **Species** | **Duration of burial (mths)** | **Season in the field** | **Summer incubation temperatures** | **Autumn/spring incubation temperatures** | **Winter incubation temperatures** |
| --- | --- | --- | --- | --- | --- |
| *B. anemonifolia* | 0 | summer | C, H, S, HS | C, H, S, HS | C, H, S, HS |
|  | 4 | winter | C, H, S, HS | C, H, S, HS | C, H, S, HS |
|  | 7 | spring | C, H, S, HS | C, H, S, HS | C, H, S, HS |
|  | 10 | summer | C, H, S, HS | C, H, S, HS | C, H, S, HS |
|  | 13 | autumn | C, H, S, HS | C, H, S, HS | C, H, S, HS |
| *B. floribunda* | 0 | summer | C, H, S, HS | C, H, S, HS | C, H, S, HS |
|  | 4 | winter | C, H, S, HS | C, H, S, HS | C, H, S, HS |
|  | 7 | spring | C, H, S, HS | C, H, S, HS | C, H, S, HS |
|  | 10 | summer | C, H, S, HS | C, H, S, HS | C, H, S, HS |
|  | 13 | autumn | C^1^, H^1^, S^1^, HS^1^ | C, H, S, HS | C, H, S, HS |
|  | 16 | winter |  |  | S, HS |
|  | 19 | spring |  | S | S, HS |
|  | 22 | summer | S |  | S, HS |
|  | 25 | autumn |  | C, S | S, HS |
| *B. fraseri* | 0 | summer | C, H, S, HS | C, H, S, HS | C, H, S, HS |
|  | 4 | winter | C, H, S, HS | C, H, S, HS | C, H, S, HS |
|  | 7 | spring | C, H^2^, S, HS^2^ | C, H^2^, S, HS^2^ | C, H^2^, S, HS^2^ |
|  | 10 | summer | C, H, S, HS | C, H, S, HS | C, H, S, HS |
|  | 13 | autumn | C^1^, H^1,2^, S^1^, HS^1,2^ | C, H^2^, S, HS^2^ | C, H^2^, S, HS^2^ |
|  | 16 | winter | H, HS |  | HS |
|  | 19 | spring | H, HS | H, HS |  |
|  | 22 | summer | H, HS |  |  |
|  | 25 | autumn | H, HS | C, H, HS |  |
| *B. ledifolia* | 0 | summer | C, H, S, HS | C, H, S, HS | C, H, S, HS |
|  | 4 | winter | C, H, S, HS | C, H, S, HS | C, H, S, HS |
|  | 7 | spring | C, H^2^, S, HS^2^ | C, H^2^, S, HS^2^ | C, H^2^, S, HS^2^ |
|  | 10 | summer | C, H, S, HS | C, H, S, HS | C, H, S, HS |
|  | 13 | autumn | C^1^, H^1,2^, S^1^, HS^1,2^ | C, H^2^, S, HS^2^ | C, H^2^, S, HS^2^ |
|  | 16 | winter | H, HS |  | HS |
|  | 19 | spring | H, HS | H, HS |  |
|  | 22 | summer | H, HS |  |  |
|  | 25 | autumn | H, HS | C, H, HS |  |
| *B. pinnata* | 0 | summer | C, H, S, HS | C, H, S, HS | C, H, S, HS |
|  | 4 | winter | C, S | C, S | C, S |
|  | 7 | spring | C, S | C, S | C, S |
|  | 10 | summer | C, S | C, S | C, S |
|  | 13 | autumn | C, S | C, S | C, S |
|  | 16 | winter | S |  | S |
|  | 19 | spring | S | C, S |  |
|  | 22 | summer | S |  |  |
|  | 25 | autumn | S | C, S |  |
| *B. serrulata* | 0 | summer | C, H, S, HS | C, H, S, HS | C, H, S, HS |
|  | 4 | winter | C, H, S, HS | C, H, S, HS | C, H, S, HS |
|  | 7 | spring | C, H, S, HS | C, H, S, HS | C, H, S, HS |
|  | 10 | summer | C, H, S, HS | C, H, S, HS | C, H, S, HS |
|  | 13 | autumn | C^1^, H^1^, S^1^, HS^1^ | C, H, S, HS | C, H, S, HS |
|  | 16 | winter |  | HS | S |
|  | 19 | spring |  | S, HS | S |
|  | 22 | summer | S | HS | S |
|  | 25 | autumn |  | C, S | S |
| *B. thujona* | 0 | summer | C, H, S, HS | C, H, S, HS | C, H, S, HS |
|  | 4 | winter | C, S | C, S | C, S |
|  | 7 | spring | C, S | C, S | C, S |
|  | 10 | summer | C, S | C, S | C, S |
|  | 13 | autumn | C^1^, S^1^ | C, S | C, S |
|  | 16 | winter |  | S | S |
|  | 19 | spring |  | C^,^ S | S |
|  | 22 | summer | S | S | S |
|  | 25 | autumn |  | C, S | S |

^1^Treatment interrupted in week 3 by incubator failure

^2^Treatment corrupted / data excluded
