# Supplementary material for: Fire Seasonality, Seasonal Temperature Cues, Dormancy Cycling, and Moisture Availability Mediate Post-fire Germination of Species With Physiological Dormancy
Source: Front Plant Sci. 2021 Dec 3;12:795711. doi: 10.3389/fpls.2021.795711 (PMC8678276; doi:10.3389/fpls.2021.795711)
Supplement: Supplementary file 1 [file Data_Sheet_1.zip › Supplementary Material 2 - Data imputation.docx]

Supplementary Material

**Fire seasonality, seasonal temperature cues, dormancy cycling, and moisture availability mediate post-fire germination of species with physiological dormancy**

Berin D. E. Mackenzie*, Tony D. Auld, David A. Keith and Mark K. J. Ooi

* Corresponding Author: [berin.mackenzie@environment.nsw.gov.au](mailto:berin.mackenzie@environment.nsw.gov.au) (B. D. E. Mackenzie)

**Supplementary Material 2. Imputation of missing dormancy data due to incubator failure.**

An incubator malfunction during the germination trials of seeds buried for 13 months resulted in loss of data on dormancy alleviation in *Boronia ledifolia* and *B. fraseri* between 4–14 weeks’ incubation. Appropriate estimates were imputed for each species based on observed patterns throughout the two-year trial, as described below.

***Boronia ledifolia***

*Supporting information*

1. Maximum germination of *B. ledifolia* (a minimum estimate of dormancy alleviation) is almost always observed in response to the combined heat pulse plus smoke treatment (HS) for this species (Supplementary Figure 1).
2. The response to HS at summer temperatures is almost always slightly greater than or equal to that to HS at autumn temperatures (Supplementary Figure 2), but differences in responses to HS at summer versus autumn incubation temperatures are negligible after 6 weeks’ incubation.

*Imputation solution*

Germination responses to HS at autumn temperatures were used as *minimum* estimates of the likely responses to HS at summer temperatures and the *minimum* extent of dormancy alleviation after 13 months’ burial between 4–14 weeks’ incubation in Figures 4 & 5 of the main text (data on germination responses to HS at summer temperatures are available up to week 3).

***Boronia fraseri***

*Supporting information*

1. The minimum time to onset of germination for *B. fraseri* across all treatments throughout the two-year trial was 4 weeks.
2. Maximum germination of *B. fraseri* (a minimum estimate of dormancy alleviation) is almost always observed in response to HS, especially during the second year of the study (Supplementary Figure 1).
3. The germination response to HS at summer temperatures is usually much greater than that to HS at autumn temperatures (Supplementary Figure 2), especially from 10 months of burial onwards, and is independent of incubation duration.
4. Unlike in *B. ledifolia*, responses of *B. fraseri* to HS at autumn temperatures are likely to provide a poor indication of those to HS at summer temperatures.
5. A significant release of dormancy was observed after 13 months’ burial (the second summer in the soil seed bank; see increase in germination in response to HS, (Supplementary Figure 1). Following this release, the response to HS at summer temperatures ranged from 24.7% (19 months’ burial/second spring in the soil seed bank) to 56.2% (16 months’ burial/second winter in the soil seed bank).

*Imputation solution*

The range of responses to HS at summer temperatures between 16–25 months’ burial was used to determine plausible minimum estimates of the likely responses to HS at summer temperatures and the *minimum* extent of dormancy alleviation after 13 months’ burial between 4–14 weeks’ incubation in Figures 4 & 5 of the main text (data on germination responses to HS at summer temperatures are available up to week 3).


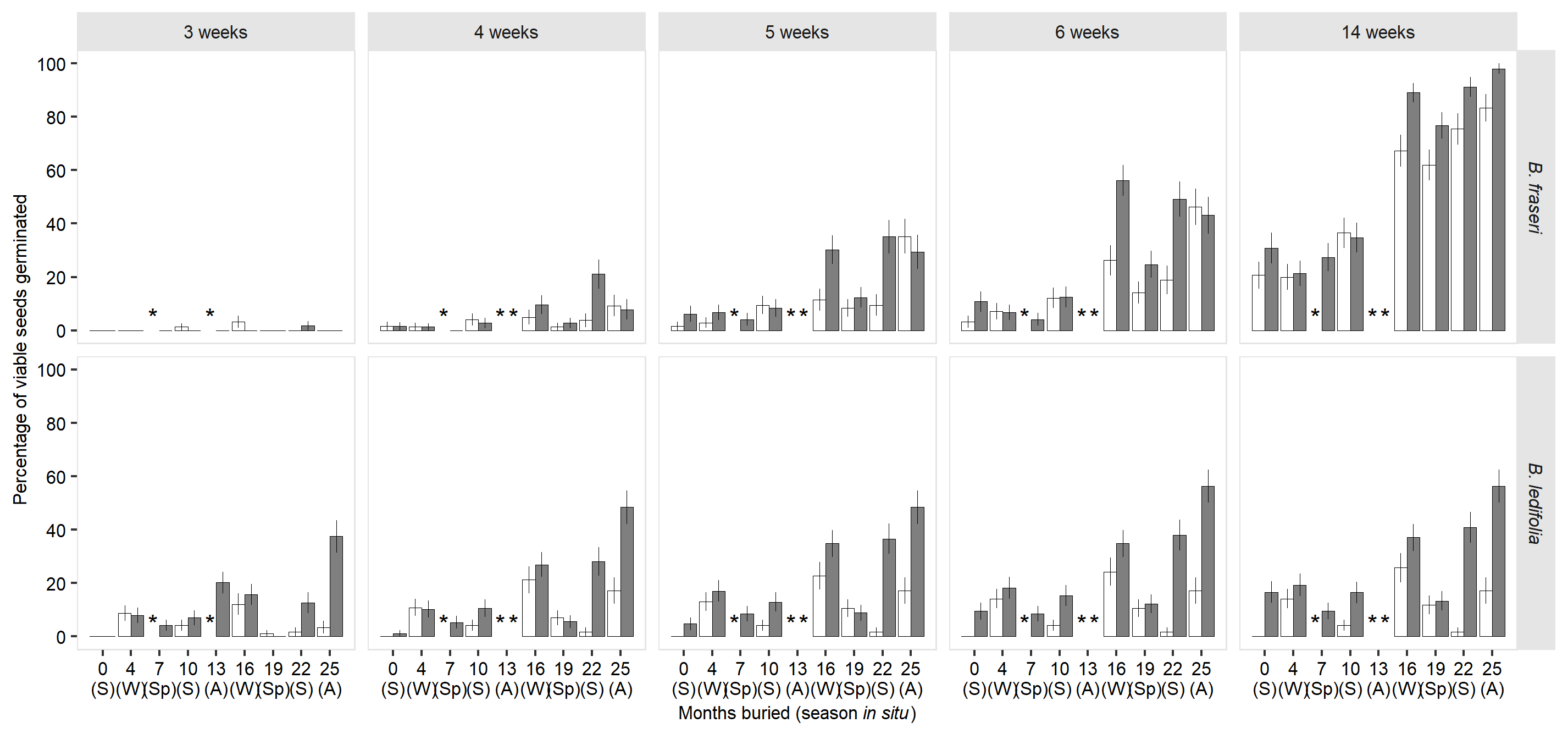


**Supplementary Figure 1.** Relative efficacy of the heat pulse treatment (white bars) and the combined heat pulse plus smoke treatment (grey bars) at maximising germination and estimating the minimum proportion of dormant seeds for *Boronia fraseri* and *B. ledifolia* (S – summer; A – autumn; W – winter; Sp – spring). * – data on heat pulse responses (white bars) unavailable at 7 and 13 months’ burial, and data on the combined heat pulse plus smoke unavailable at 13 months burial after 3 weeks’ incubation.


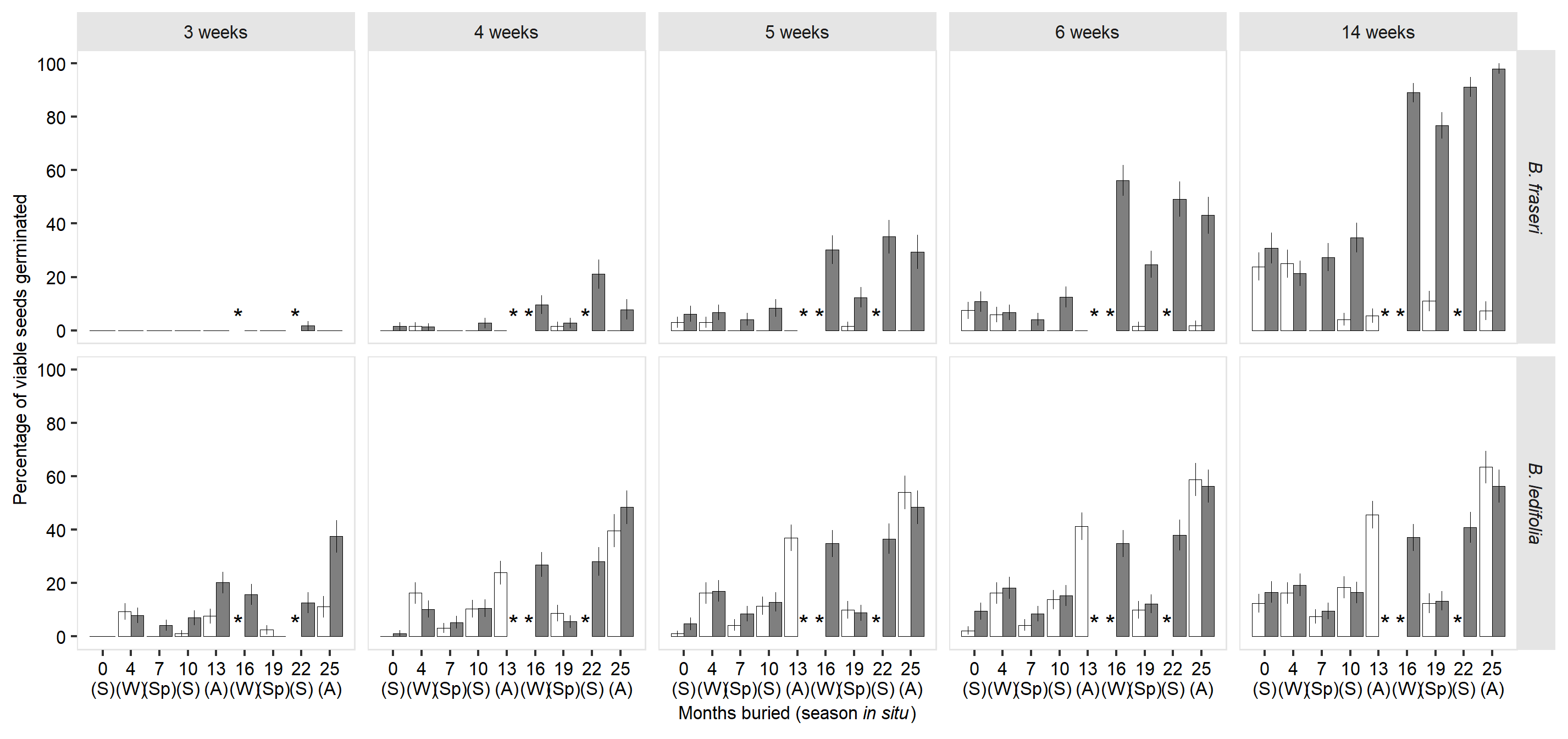


**Supplementary Figure 2.** Relative effect of summer (white bars) versus autumn (grey bars) incubation temperatures on maximum germination (minimum dormancy alleviation) in response to the combined heat pulse plus smoke treatment for *Boronia fraseri* and *B. ledifolia* (S – summer; A – autumn; W – winter; Sp – spring). * – data not available due to incubator failure (13 months’ burial) or treatment not applied (other timesteps).
