# Supplementary material for: Fire Seasonality, Seasonal Temperature Cues, Dormancy Cycling, and Moisture Availability Mediate Post-fire Germination of Species With Physiological Dormancy
Source: Front Plant Sci. 2021 Dec 3;12:795711. doi: 10.3389/fpls.2021.795711 (PMC8678276; doi:10.3389/fpls.2021.795711)
Supplement: Supplementary file 1 [file Data_Sheet_1.zip › Supplementary Material 3 - Soil moisture.docx]

Supplementary Material

**Fire seasonality, seasonal temperature cues, dormancy cycling, and moisture availability mediate post-fire germination of species with physiological dormancy**

Berin D. E. Mackenzie*, Tony D. Auld, David A. Keith and Mark K. J. Ooi

* Corresponding Author: [berin.mackenzie@environment.nsw.gov.au](mailto:berin.mackenzie@environment.nsw.gov.au) (B. D. E. Mackenzie)

**Supplementary Material 3. Seasonal variation in soil moisture availability.**

Rainfall in the study region (Sydney, southeastern Australia) is aseasonal (Figure 2, main text) and an infinite number of hydration and dehydration scenarios for soils and buried seeds may eventuate in the field. In general, in this region and other temperate regions, evapotranspiration declines and is more homogeneous in the cooler months (Supplementary Figure 3) and this promotes soil moisture retention from any precipitation inputs that occur immediately prior to, or during, these periods (soil moisture is inversely related to evapotranspiration). In the warmer months, however, increases in soil moisture with precipitation inputs are more transient due to higher (and more heterogeneous) evapotranspiration, especially in the topsoil where most seeds are stored (i.e., the top 5–10 cm; e.g., Auld & Denham, 2006). Seasonal variation in soil moisture occurs throughout temperate climates (e.g. Figure 3 of Bradstock & Bedward, 1992), but is especially pronounced in those with summer droughts, such as Mediterranean-type climates (e.g. Figure 1 of Merritt, Turner, Clarke, & Dixon, 2007). Occasional years of exceptionally high rainfall over the warmer months may increase soil moisture persistence and diminish ‘germination interval squeeze’.


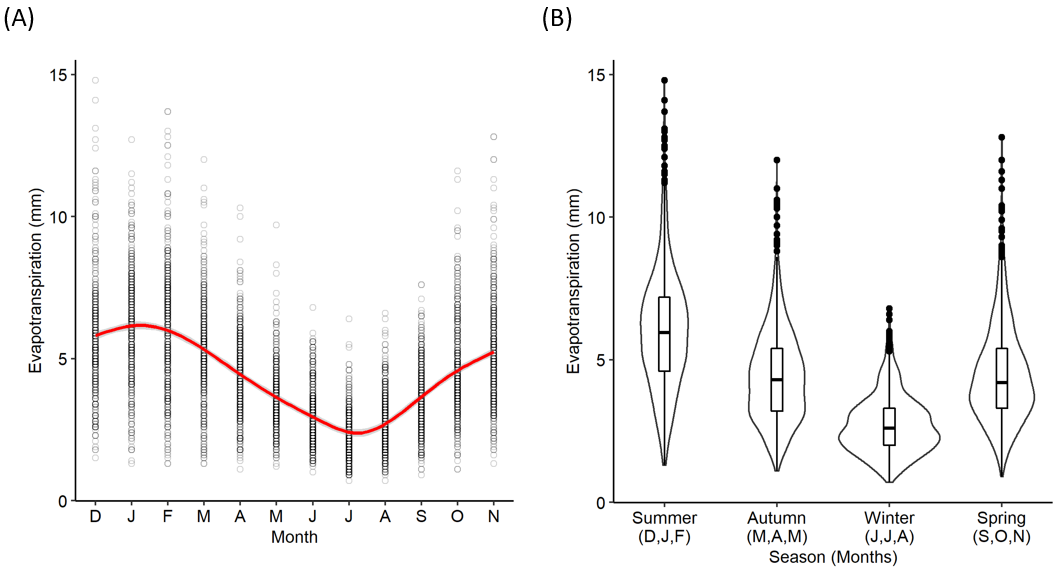


**Supplementary Figure 3.** Monthly **(A)** and seasonal **(B)** trends in evapotranspiration in the Sydney region of southeastern Australia. Data are from the Sydney Airport AMO weather station for the period 1^st^ January 2009 to 28^th^ October 2021, courtesy of the Australian Government Bureau of Meteorology (2021).

**References**

Auld, T. D., and Denham, A. J. (2006). How much seed remains in the soil after a fire? *Plant Ecol.* 187, 15–24.

Australian Government Bureau of Meteorology (2021). Climate Data Online. Available at: http://www.bom.gov.au/climate/data. Accessed 30^th^ October 2021.

Bradstock, R. A., and Bedward, M. (1992). Simulation of the effect of season of fire on post-fire seedling emergence of two Banksia species based on long-term rainfall records. *Aust. J. Bot.* 40, 75. doi:10.1071/BT9920075.

Merritt, D. J., Turner, S. R., Clarke, S. W., and Dixon, K. W. (2007). Seed dormancy and germination stimulation syndromes for Australian temperate species. *Aust. J. Bot.* 55, 336–344. doi:10.1071/BT06106.
